# The role of statins in patients with early diabetic nephropathy: A protocol for systematic review and meta-analysis

**DOI:** 10.1097/MD.0000000000029099

**Published:** 2022-06-17

**Authors:** Xi Zhao, Shu Chun Zhou, Xiu Fang Wang, Hong Wu Liao

**Affiliations:** Health School of Nuclear Industry, The Affiliated Nanhua Hospital, Hengyang Medical School, University of South China, Hengyang City, Hunan Province, China.

**Keywords:** diabetic nephropathy, meta-analysis, protocol, statins, systematic review

## Abstract

**Background:**

Little is known about the renoprotective effects of statins on the regulation of urinary oxidative stress markers and proteinuria in patients with diabetic nephropathy. Therefore, we conducted this protocol of systematic review and meta-analysis to evaluate the role of statins in patients with early diabetic nephropathy.

**Methods:**

We followed the Preferred Reporting Items for Systematic Reviews and Meta-Analyses Protocols reporting guidelines to conduct this study. The electronic databases EMBASE, PUBMED, CINAHL, and Web of Science will be searched from the earliest available time to July 2022. The population is defined as participants with early diabetic nephropathy. The Intervention groups are given any one of the statins, such as simvastatin or rosuvastatin. The control groups are treated with angiotensin-converting enzyme inhibitor or placebo alone. The primary outcome is estimated glomerular filtration rate; secondary outcome is serological indicators including triglyceride, cholesterol, C-reactive protein, and complications. The Jadad scale will be used to assess the methodological quality of each study included in this meta-analysis.

**Result & Conclusion:**

We hypothesized that statins would have a positive renoprotective effect in such patients.

**OSF registration number:**

10.17605/OSF.IO/ESMWR.

## 1 Introduction

More than 80% of people with diabetes have type 2 diabetes, which is characterized by slow onset, heterogeneous disease, and the influence of environmental factors and polygenic inheritance.^[1,2]^ Type 2 diabetes is the leading cause of chronic kidney disease and end-stage renal disease. Diabetic nephropathy, a common complication in diabetic patients, is rising with the rapid increase in the prevalence of type 2 diabetes. The early stages of diabetic nephropathy are characterized by microalbuminuria, whereas as diabetic nephropathy progresses, macroalbuminuria develops with marked renal impairment.^[3,4]^ Therefore, early treatment of diabetic nephropathy patients is crucial to alleviate renal impairment and reduce renal-related mortality.

Dyslipidemia is of interest as a known factor that may contribute to worsening renal function and cardiovascular disease in patients with type 2 diabetes. Statins have been found to be effective in improving blood lipid parameters, such as lowering low-density lipoprotein cholesterol and triglycerides, and raising high-density lipoprotein cholesterol.^[5–7]^ Meanwhile, statin therapy can effectively reduce inflammation and glomerular filtration dysfunction in patients with chronic kidney disease.^[8]^ In addition, statins exert multiple cardioprotective effects by increasing vascular nitric oxide bioavailability and reducing oxidative stress and inflammatory cytokines.^[9]^

However, little is known about the renoprotective effects of statins on the regulation of urinary oxidative stress markers and proteinuria in patients with diabetic nephropathy. Although recent studies have shown that statins can protect renal function by improving vascular endothelial function, inhibiting thrombosis, anti-oxidation, and regulating immunity. However, the sample size of such studies is small and the research quality is uneven, which cannot provide sufficient evidence-based basis.^[10–12]^ Therefore, we conducted this protocol of systematic review and meta-analysis to evaluate the role of statins in patients with early diabetic nephropathy. We hypothesized that statins would have a positive renoprotective effect in such patients.

## 2 Materials and methods

### 2.1 Study registration

We followed the Preferred Reporting Items for Systematic Reviews and Meta-Analyses Protocols reporting guidelines to conduct this study. The prospective registration has been approved by the Open Science Framework registries. Ethical approval is not necessary because the present meta-analysis will be performed based on previous published studies.

### 2.2 Search strategy and eligibility criteria

The electronic databases EMBASE, PUBMED, CINAHL, and Web of Science will be searched from the earliest available time to July 2022. The concepts of population, intervention, outcome and design are combined with the “AND” operator. The key phrases are as following: “statin,” “diabetic nephropathy,” “diabetic kidney disease” and “prospective.” The population is defined as participants with early diabetic nephropathy. The Intervention groups are given any one of the statins, such as simvastatin or rosuvastatin. The control groups are treated with angiotensin-converting enzyme inhibitor or placebo alone. The primary outcome is estimated glomerular filtration rate; secondary outcome is serological indicators including triglyceride, cholesterol, C-reactive protein, and complications. Types of included studies are randomized controlled trials. Exclusion criteria include conference abstract, letters, review articles, studies with a sample size <50, and studies with insufficient outcome data.

Two independent authors will import all articles into bibliography software and filtered duplicates, then independently screen the title and abstract of each article using pre-determined eligibility criteria. Disagreements are resolved through discussion. Differences that could not be resolved by discussion will be referred to a third reviewer for consensus. Reference lists containing articles will be manually searched and citation tracking will be applied using Google Scholar to identify any additional articles to include.

### 2.3 Data extraction

Two independent authors will extract the following descriptive raw information from selected studies: study characteristics such as authors, study design, study language, year of publication, mean follow-up period; patient demographics such as number, mean age, body mass index and sex ratio; details of interventions and outcome measures. The outcomes include estimated glomerular filtration rate, triglyceride, cholesterol, C-reactive protein, and complications. If data is missing or cannot be extracted directly, we will contact the corresponding author to ensure that the information is complete.

### 2.4 Risk of bias assessment

The Jadad scale will be used to assess the methodological quality of each RCT included in this meta-analysis. The scale consists of 3 assessment elements: randomization (0–2 points), blinding (0–2 points), dropout and withdrawal (0–1 points). Each element will be assigned one point if it is mentioned in the article and another point would be given if the method of randomization and/or blinding has been described in detail and appropriately. One point will be deducted if randomization and/or blinding methods are not appropriate, or if dropouts and withdrawals are not recorded. Scores on the Jadad scale range from 0 to 5. Articles with a Jadad score ≤2 are considered low quality. The study will be considered high quality if the Jadad score is ≥3.

### 2.5 Data analysis

Data analysis will be performed using Review Manager software for Windows (RevMan version 5.3, Copenhagen; Nordic Cochrane Centre, The Cochrane Collaboration, 2014). For continuous data, the standardized mean difference with a 95% confidence interval (95% CI) will be calculated. Dichotomous data are expressed as hazard ratios indicating the effect of the intervention. Statistical heterogeneity of the data is assessed using the *I*^2^ value and the Chi-Squared test. If *I*^2^ > 50% and *P* < .05, the statistics are considered to be heterogeneous, and a random-effects model is used. Otherwise, the fixed effects model will be performed for analysis.

## 3 Discussion

Diabetic nephropathy is a leading cause of end-stage renal disease in adults of Western countries. However, little is known about the renoprotective effects of statins on the regulation of urinary oxidative stress markers and proteinuria in patients with diabetic nephropathy. Although recent studies have shown that statins can protect renal function by improving vascular endothelial function, inhibiting thrombosis, anti-oxidation, and regulating immunity. However, the sample size of such studies is small and the research quality is uneven, which cannot provide sufficient evidence-based basis.^[10–12]^ Therefore, we conducted this protocol of systematic review and meta-analysis to evaluate the role of statins in patients with early diabetic nephropathy. We hypothesized that statins would have a positive renoprotective effect in such patients.

## Author contributions

**Conceptualization:** Xi Zhao.

**Formal analysis:** Xi Zhao.

**Funding acquisition:** Xi Zhao, Shu Chun Zhou.

**Investigation:** Xi Zhao,Shu Chun Zhou.

**Methodology:** Xi Zhao.

**Project administration:** Xi Zhao.

**Software:** Xi Zhao,Xiu Fang Wang.

**Supervision:** Xi Zhao,Hong Wu Liao.

**Writing – original draft:** Xi Zhao.

**Writing – review & editing:** Xi Zhao, Hong Wu Liao.
